# The Principles and Practice of Obstetric Medicine, in a Series of Systematic Dissertations on Midwifery and on the Diseases of Women and Children. Illustrated by Numerous Plates

**Published:** 1837-04

**Authors:** 


### Art. VII.

1. *The Principles and Practice of Obstetric Medicine, in a Series of Systematic Dissertations on Midwifery and on the Diseases of Women and Children. Illustrated by numerous Plates.* By David D. Davis, m.d. m.r.s.l., Professor of Midwifery in the London University, &c.—*London,* 1836. Two Vols. 4to. pp. 1294.

2. *Operationslehre für Geburtshelfer, in zwei Theilen.* Von Dr. Hermann Friedrich Kilian, ordentl. öffentl. Professor der Geburtshülfe und der Geburtshülflichen Klinik an der Rheinischen Friedrich-Wilhelms Universität, u. s. w. *Erster Theil: die Operative Geburtshülfe.—Bonn.* 1834, 1836. 8. 956 s.

*Instructions in Operating for Accoucheurs, in two Parts.* By Hermann Frederick Kilian, m.d., Public Professor in ordinary of Midwifery, and of the Midwifery Clinic, at the University of Bonn, &c. *Part First: Operative Midwifery.—Bonn.* 8vo. pp. 596.

The general subject of these two works being the same, the date of their publication the same, and as they both emanate from professors filling very important chairs in their respective countries, we have thought it best to join them together in one article. We shall, however, notice each separately, without reference to the other; only endeavouring, as much as possible, to avoid repetition of the same subjects, and striving to make one half of the review the complement of the other. The critical reader may draw his own conclusions respecting the relative merits of the two publications.

I. With many of the separate parts of the first work on our list, no doubt, most of our readers are acquainted. It being at length completed, we hasten to pay it that degree of attention which an undertaking so comprehensive, and emanating from such a quarter, must demand. The rank and station of the writer, his experience as a teacher, and his connexion with some of the first lying-in charities in the metropolis; the size and extent of the work, and the time occupied in its completion,—all tended to excite our expectations, and led us to look for a very perfect system; one that should constitute an established book of reference to our practitioners at home, and be regarded abroad as containing an accurate account of British practice; and thus tend to exalt or depreciate, as the case might be, in the minds of our foreign neighbours, the obstetric department of medicine in this country. Under these circumstances, therefore, we feel called upon to examine this book with a greater degree of strictness than a work appearing under less imposing auspices would demand.

Although Dr. Davis has, to a certain extent, disarmed our criticism by his modest profession, that his work contains merely "a moderately extensive view of the elements of obstetric medicine," yet we feel we should ill discharge the duty we owe our readers, if, accepting this as a sufficient apology for imperfections, we were to omit to notice any thing which should appear to us defective, or hesitate to point out what we deemed errors, either of principles or practice. The author's estimate of his performance will be best understood from his own words: he says that "he has furnished the student, by the direct instruction of his book, with the means of acquiring an accurate knowledge of the very important department of medical science of which he treats; and gentlemen already established and experienced in practice, with facilities, by its numerous references, for acquiring a more substantial and profound acquaintance with any division, or with the entire range of subjects of which it treats, than is usually considered necessary for the general purposes of the profession." This, then, must be the touchstone by which it is to be tested.

In the arrangement of his work, Dr. Davis adopts three great divisions of his subjects: the *first part* including the anatomy, physiology, and pathology of the genital organs; *the second*, pregnancy and its consequences, with the puerperal state; and *the third*, the diseases of children.

Without entering into the work at all, we cannot help being struck with the unequal space he apportions to these different subjects: to the first he devotes 815 pages, to the second 393, and to the last but sixty pages. Indeed, Dr. Davis himself appears to have foreseen the objections likely to be raised to the manner of his arrangement in this respect, as he apologises for "the subjects of his work not having received that uniform equality of illustration to which a treatise of this kind might have possibly laid claim." We confess we think that the author would have better executed his task had he gone deeper into some of his practical points, and had, in other instances, avoided wandering into merely curious discussions, or relations of isolated cases of very rare diseases. We have an example of this kind of digression in his account of diminution and excess observed in the hairy covering of the mons veneris, where we have two pages (35, 36,) drawn from the ephemerides, Buffon, and M. Chabert, the fire-king, of very questionable, and certainly extraneous matter; while, on the other hand, he discusses the subject of pruritus in a few lines, and omits even an enumeration of several of the varied states of disease of which it is symptomatic.

The second chapter contains a brief general description of the fœtal, young, and adult pelvis; with a parallel between the pelves of the sexes, an explanation of the measurements and diameters of the pelvis, and a disquisition on the use of pelvimeters. Our author maintains, what all men of experience must admit, that the accoucheur's finger and hand are the best pelvimeters; but we cannot assent to all the recommendations in the following passage:

"The point of the forefinger is to be carried to the promontory of the sacrum, and made to rest on the sacral projection, whilst the portion of it that crosses the arch of the pubis should be accurately marked with the nail of the index-finger of the practitioner's other hand. The intermediate distance between the finger so indented and its point resting on the promontory of the sacrum, will, of course, be the measure of a line drawn from the promontory of the sacrum to the apex of the arch, the thickness of the symphysis pubis inclusive. Making an allowance for this thickness, and for the greater length of the line thus obtained, by reason of its greater inclination than of the conjugate diameter, the length of the latter line, the conjugate diameter of the brim of the pelvis, is determined with considerable accuracy. Should the practitioner's index-finger prove too short to reach the promontory of the sacrum, presuming on its being of the ordinary length, and on its being passed up in a proper direction, it would then be a matter of generally safe inference that the pelvis was of sufficient capacity in the direction of the conjugate diameter. To ascertain the existence of room on either side, the whole hand is to be introduced into the vagina, and carried up edgewise, with the index-finger in front, and the little and ring fingers behind, to the lateral portions of the pelvic cavity, so that the points of the fingers shall rather more than clear the brim. Should there be found sufficient room on either side of the diametrical line for the hand so introduced to maintain the parallelism of its fingers,—i. e. to be there without being obliged to ride over each other,—then it would be a matter of conclusion that, with sufficient space in the middle, added to the ordinary space on either side, there would be found ample room for the descent of the child's head into the general cavity. The practitioner then would have to pass his hand along the other side of the pelvis, and subject its dimensions to the same test. If indeed the hand was carried up along and nearly in contact with the lateral parietes of the pelvis, and room was found there for three fingers to be together in moderate parallelism, the conjugate diameter being at the same time of sufficient length, it would be competent for the medical attendant, as a general rule, to infer favorably of the dimensions of such a pelvis. The transverse diameter of the outlet may be accurately enough ascertained for practical purposes, by merely introducing the hand into the pelvis, and placing the thickest and broadest part of it transversely across the vulva, so as to occupy the line of the short diameter. The breadth of an average sized hand across the metacarpal joints is very nearly three inches and a half. A pelvis, therefore, sufficiently large to permit the hand to occupy easily, or even without sustaining any serious pressure, the lateral boundaries of the outlet, the position just described, would be sufficiently ample for the safe passage through it of a well-grown fœtal head at the full period of gestation." (P. 21-23.)

We conceive that the directions for introducing the *hand* so freely into the vagina are both unnecessary and improper; and, indeed, this practice can never be generally tolerated. Examinations with the finger must be *made* sufficiently correct to give a right result.

We have long been of opinion that, in Dr. Hull's theory of the distinctive deformities produced in the pelvis by rickets and mollities ossium, he had generalized far more than the true nature of the case warranted: we have, in our own collection, preparations which justify us in arriving at this conclusion; preparations exhibiting the triangular figure which he ascribed to mollities ossium, but which we know to have been the result of rickets. We feel no hesitation, then, in coinciding so far with Dr. Davis in his opposition to this doctrine, although we may hesitate in admitting with him that the pelvis, after rickets, possesses the power of regaining a very fair share of symmetry. (P. 32.)

In Dr. Davis's anatomical descriptions, he is sufficiently minute without being tiresome. He has, however, fallen into an error in the description of the locality of the urethral orifice, which able anatomists have fallen into before him: he has described this aperture as midway between the clitoris and the anterior edge or commissure of the vulva; whereas, in the natural state of parts, it is much closer to the edge of the vulva than to the clitoris. What has, no doubt, given rise to this mistake is the fact that the vaginal margin at this part is sometimes full and prolonged, either from relaxation, congestion, or some other cause.

In treating of the diseases of the labium, our author does not object to incisions in cases of infiltration of this organ; and recommends stimulating in preference to soothing applications for the treatment of abscess in it. He reports (p. 52,) a case of protrusion of the urinary bladder into the left labium, which was seen in company with Mr. Lawrence. If a more accurate investigation of this case had proved it to be the bladder that constituted "this cyst containing watery fluid," we should have esteemed the case as one of extreme interest: we are not, however, satisfied that the data, at least as given by Dr. Davis, quite authorize this conclusion; and what tends to increase the doubt is the author's own statement, that "no urine could be forced out of the urethra by pressing the tumour with the hand, nor by that means could any sensible reduction be made in its body." In a case of this avowedly doubtful character, and more particularly when another eminent surgeon had arrived at a different conclusion, as to its nature, from that of the author and Mr. Lawrence, the public should have been furnished with more accurate means of forming a true estimate of the case. There is no mention made of any attempt to trace the tumour to the seat of the bladder, nor explanation as to the exact place where it escaped.

The observations on Varix are interesting, and well worth the reader's attentive perusal.

Sixteen pages are very profitably occupied in discussing Syphilis and Syphiloid Diseases, as they attack the female. They are treated under three heads,—Inflammation of the genital surfaces, with discharges; gonorrhœa and warty fungoid excrescences; and ulcers. Dr. Davis evinces that he is a decided mercurialist; and, in advocating the mercurial treatment, makes a statement which certainly does not correspond with our experience on this subject,—namely, that he never has seen secondary symptoms occur, or syphilitic children given birth to, after full courses of mercury.

The author has been at much pains to investigate some of the more rare female diseases, which hitherto have attracted but little attention from our practical obstetric authors. We have an example of his success in this respect in his observations on carunculous or hydatid growths in the urethra and bladder; in the treatment of which he has recommended the introduction of a cylindrical instrument, made of ivory, with a bulbous extremity; calculated, by the pressure it produces, to destroy the fungoid growth.

In those embarrassing cases of contraction or banded state of the vagina, the effect of inflammation most commonly produced in a former delivery, the accoucheur is well aware of the benefit derived from incising the bands with a bistoury, and that this simple process often prevents the occurrence of most extensive lesions and lacerations, both in the recto-vaginal septum, perineum, vagina, and uterus. To our astonishment, Dr. Davis condemns this practice, and appears to rely altogether, in such cases, on the use of the lancet. Depletion we esteem as a most important adjunct in the case in question, where depletion is admissible; but we would warn the young practitioner, if he values the safety of his patient, to be cautious how he adopts the recommendation of omitting to divide these bands; and we do this with a firm and painful conviction pressing upon us of the mischiefs that have occurred in our own practice from such an omission. Dr. Davis gives a case of adherent walls of the vagina, in connexion with this subject, with accumulated menstruous secretion beyond the point of union, in which the patient was resigned to her fate. Surely, some operation might have been undertaken in a case of this kind, in the present state of surgical and obstetric science. He also reports an unaccountable but most interesting case of spontaneous membraneous formation, occurring in a very short time after sloughing of the recto-vaginal septum, and completely closing up the aperture. His account of sloughing of the bladder, with vesico-urinary fistula, is very imperfect; and no notice whatever is taken of the benefits to be derived from the use of the actual cautery, as recommended and practised by Dupuytren in these distressing cases.

The practice recommended by Dr. Davis of free general depletion in cases of vaginal inflammation, the effect of pressure of the head in labour, we esteem as more than objectionable; and, if generally acted on, we have no hesitation in stating that the most injurious effects must result from it. Every practitioner is aware that the strongest tendency exists, in inflammation of this organ at this time, to run rapidly into slough and gangrene, and that the great difficulties he has to contend with are the prostration and exhaustion which occur: are these, then, let us enquire, likely to be guarded against or benefited by free depletion? But it may be argued, that if depletion was freely and sufficiently early had recourse to, the gangrenous tendency would be checked or prevented. In answer to this we would say, that those who have watched these cases attentively well know that the mischief is done before the delivery is effected, and that, so far from reducing our patients afterwards by depletion, our energies are, from the train of symptoms present, required imperatively to be directed towards maintaining their strength by such means as are safe and admissible.

Dr. Davis's remarks on the subject of Entero-vaginal Hernia are judicious.

"It might be very possible for a practitioner, not much experienced in matters of this kind, to mistake, for instance, a case of hernial descent for prolapsion of the vagina. The practical result, of course, would be, that he would content himself with merely introducing a pessary into the vagina, without previously effecting the reduction of the hernia; and of such a blunder the consequences, it is obvious, might be very serious."

We have ourselves known strangulation of the intestine to occur unsuspected under such circumstances; and Professor Guntz (*De Hern. Libell.*) relates a case in which the attending surgeon destroyed his patient by plunging a bistoury into a hernial tumour, under the impression that it was an abscess. From the comparative rarity of this disease, it is not well understood by the profession at large.—Garengeot's case, recorded in the *Mem. de l'Acad. Roy. de Chir.*, t. i., was the first that drew the attention of medical men to the subject. It occurs only at the anterior and posterior part of the vagina, and is generally observed as the result of effort when the parts are in a state of relaxation after delivery. Boyer treats of the possibility of hernia occurring by the separation of certain of the vaginal fibres of the external tunic, the protruded gut effecting the distention of the internal tunic only. Dr. Davis reports a case of this nature, attended by himself and Dr. Sims, replete with interest; and, although he occupies seven pages in the recital, we were gratified and pleased with every line of it. The sound practice adopted, and the happy results under the most unpromising circumstances, render it a case particularly meriting the attentive perusal of all obstetricians. The accident did not occur, as it usually does, after pregnancy. The patient was freely depleted; great care was taken in effecting, as soon as possible, the reduction of the tumour, and retaining it, when reduced, by the application of a properly adapted pessary, and by guarding the horizontal posture for a sufficient time. The patient was perfectly cured, and there is every reason to suppose that the separated fibres were reunited.

In leaving this subject, upon which we certainly should have quoted our author at length, had we not such a crowd of other matter presenting itself to us for notice, we must say that we look upon the observations upon entero-vaginal hernia and cystocele as among the best contained in the work. The accurate and discriminating attention devoted to its diagnosis, the instructive cases recorded, the judicious quotations, (if we except the extreme length and repetitions in one case,) and the sound practice adopted, whether connected or unconnected with delivery, fully justify our good opinion; and we refer our readers to the original with a firm conviction that they will derive from it both gratification and instruction.

Dr. Davis's account of menstruation is very interesting, although we cannot avoid feeling a little provoked at the space devoted by the author to combat such absurd theories as that of Roussel, who denied menstruation being a function with which the human female was originally invested, ascribing it, as our readers are no doubt aware, to an effort on the part of the vessels of the womb, to relieve the circulation from a repletion, the effect of luxurious living, such as we see in hemorrhoidal discharges or hemorrhages in the male. No doubt, also, by some, who take a hasty view of the subject, he will be pronounced credulous in the opinions he supports with regard to serotine menstruation. It is but too much the practice with professional men to deny the possibility of the existence of any physiological or pathological matter, because its occurrence may happen to be opposed to their preconceived ideas, or may not have occurred within the sphere of their observation, however restricted this may have been. Dr. Davis, however, coolly, and (we think) philosophically, enquires into this subject. He weighs the testimony of those who have treated of it; he examines the reported cases, and enquires how the symptoms and states hinge upon each other, in establishing or invalidating their accuracy. Whilst he admits that an immense majority of the cases of sanguineous uterine discharges, recorded as sustained by women in advanced life, are the result of diverse diseases; yet, from the authority on which many of the cases rest, and from the subjects being hale, vigorous women; from their first menstruation having generally occurred in an advanced period of their growth; from their having lived in country districts, on homely fare, and being actively occupied; from the discharge having regularly and uninterruptedly recurred, from youth to old age, &c.; our author is of opinion, (and in this we agree with him,) that it is more easy to suppose that women might live and enjoy vigorous health to an advanced period of life, subject to the condition of the protracted performance of a natural function, than that they should survive many years, and enjoy vigorous health, subsequently to their becoming the subjects of frequently recurring hemorrhages; or, in other words, of a positively diseased state of the uterus, usually attended with extreme danger even to life itself.

In the portion of his work allotted to the consideration of the Influence of Menstruation on the Health, &c., our author evinces a strong anxiety to prove that the cessation of the menstrual secretion is attended with no peculiar risk towards the female. This opinion he founds principally on Finlaison's Reports. The statistical information possessed on this, as on most other physiological subjects, is still very much in its infancy, and very contradictory results have been arrived at upon it. Thus, although Mr. Finlaison, in his table of the comparative proportion of male and female mortality kept at Ostend, arrives at the conclusion that, between the ages of forty and forty-five, only 7,094 females die for 11,107 males; yet, in the calculation of the rate of mortality of the government annuitants, during the same epoch, from forty to forty-five, 6,959 males die for 6,889 females: but, again, the excess is in favour of the female mortality, according to Quetelet's and Smit's returns, in the country districts of Belgium; for here, between forty and forty-five years of age, 8,536 females die for 5,975 males. With this extreme uncertainty and contradiction in statistics, then, we feel that, as yet, the conclusions arrived at by our author are not justifiable; nor do we think that, even upon his own showing, and granting the correctness of Mr. Finlaison's reports, he has made out his case. Suppose the greater mortality of males at the age corresponding to what has been hitherto esteemed the critical age of female life were established, we are fully aware that there may, and do, exist, in the physical constitution of the male, causes explaining this excess of mortality in him also at this period. Neither does the statement that there is no increase in the proportion of mortality in the female at this epoch, compared with the other periods of female life, go to disprove the danger attending it in the female; as she is now freed from the influences peculiar to her previous state of constitution,—as childbearing, and morbid uterine and menstruous functions. For a sufficiently appalling catalogue of the morbid conditions incident to mere disordered menstruation, we cannot go beyond that furnished by Dr. Davis himself, whilst treating on this subject, at page 298.

In our author's anxiety to establish a parallel between menstruation in the human female, and the œstrum of the lower animals, he rather involves himself in a dilemma. In a preceding part of his work, his efforts were directed to prove that the menstrual secretion was a natural and periodic relief of a state of vascular congestion or phlogosis, and that a period of time equal to the interval between two menstruous flows was necessary to effect the perfect reestablishment of this state of plethora. Now, the œstrum is the period of phlogosis, or congestiveness, at its height; and it is during this, as is well known, that in animals impregnation always occurs; whereas, the human female is most liable to impregnation when, according to Dr. Davis's already-expressed opinion, there must be the greatest want of phlogosis, viz. just after the local plethora has been relieved by the completion of the menstruous discharge. If the human female had been most liable to impregnation just before the occurrence of the menstruous flow, then would Dr. Davis's parallel have been perfect: as it happens, however, to be directly the reverse, and our author will not give up his analogy, he smooths off his difficulties by stating that, after menstruation, a sufficient amount of vascular congestiveness and phlogosis continues to ensure the presence of the required susceptibility of impregnation! And, as if determined to carry through this self-evident contradiction with a bold hand, he records his opinion in most imposing capitals.

In the treatment recommended by our author for Amenorrhœa, as well as Menorrhagia and Leucorrhœa, we feel little hesitation in pronouncing him much too indiscriminate in his administration of mercury: we also question much the judgment of his exhibiting so "full a charge of it" as he recommends. And this we do without denying its applicability as a remedial agent in certain properly selected cases. Its administration has been attended with benefit in the hands of other physicians; it was used, we are told, incidentally as a purgative, in a case of amenorrhœa, so far back as 1685, and was found to produce an immediate flow of the menses. Its efficacy in this respect is also proved in a case reported in the seventh volume of Simmons' Medical Journal; and the authority of Pitcairn, Heister, &c. is referred to, as justifying its use in leucorrhœa. Two out of the three cases which Dr. Davis reports, in proof of its efficacy in amenorrhœa, appear to us far from conclusive upon this point; several months having elapsed in both before the reestablishment of the uterine functions; whilst other and avowedly powerful emmenagogues were had recourse to in the interval. By submitting the first of these ladies to a course of mercury, and keeping up "a mild salivation for four months," which produced, as we might have expected, such a state of the gums and teeth as to require "the services of the dentist," as well as by the use of cinchona, columba, and decoction of the woods, her health was so far established that her mother pronounced her looking as well as she had done for many years, and she acquired a very good share of appetite.

"After a residence, however, of five months in the metropolis," continues Dr. Davis, "she experienced no sexual change, nor even any perceptible intimation of a return of her menses. From London she went to Tunbridge Wells, where she remained about ten weeks. Whilst there, *she took six ounces daily of a mixture consisting of five parts of Griffith's tonic mixture and three of compound decoction of aloes.* Before she left the place she menstruated sparingly once, and from that time forward she entirely ceased to be the subject of her former ailments, and rapidly became possessed of a greater degree of health and strength than she ever possessed before. The catamenial function was perfectly reestablished."

The other case adduced by Dr. Davis, in proof of the utility of mercury, is scarcely less equivocal. In it he "suggested the propriety of making a trial of the remedy exhibited with such advantage in the case just related."

"The mouth was made slightly sore by three doses of calomel and opium, in the proportion of four grains of the former to one and a half of the latter. The influence of the remedy was kept in the mouth and in the system for four entire months. At the end of that period the patient considered herself in many important respects improved in her health. The catamenial function was not, however, satisfactorily established until *after* several months *subsequently.* When that long-wished-for event happened, the *patient was taking, in considerable doses, the compound mixture of iron and the compound decoction of aloes.*"

Now, whilst we are free to admit that a third case, which is reported, may have been benefited by the mercurialization, we must say that we cannot conceive how any unprejudiced person could ascribe the reestablishment of the uterine functions in the other two cases to the mercury so perseveringly administered. We dwell not upon these cases with a view to put Dr. Davis out of conceit with a favorite plan of practice, but because we foresee the baneful effects likely to result from the adoption of his views by the junior or inexperienced members of the profession. A great opprobrium in obstetric medicine has been the empirical treatment hitherto adopted, and unnecessarily practised, in cases of amenorrhœa. It has been the object of our best practical writers and teachers, to inculcate the necessity of abstaining from unnecessary or mischievous interference in these cases, and to establish enlightened and philosophic views by examining the functional derangements of the uterus in relation to their effects as well as causes, and to treat them, as we do other diseases, upon well-defined physiological and pathological principles. Under such circumstances, we cannot but fear that, when a writer of Dr. Davis's rank and character comes forward with (to use his own words,) his "one great emmenagogue,—perhaps the greatest actually existing,—mercury," and recommends such a medicine to be administered so unsparingly, and with so little discrimination, he may do an infinity of mischief. He certainly states that judgment and discrimination are necessary in its administration; he also alludes to the treatment being discreetly and skilfully managed; but he at the same time tells us that it is "the greatest emmenagogue actually existing;" "that he does not think much of the restricted mode of administering mercury;" "that it is idle to hope for benefit without the excitement of some fever;" "that it must be exhibited in sufficient quantity to produce ptyalism, and a perceptible soreness of the mouth;" and that the average period of this *mild* constitutional affection should extend at least fourteen or fifteen weeks.

"This powerful agent," says Dr. Davis, "is only proper in chronic cases, where the balance of the different actions of the body is become much disturbed, and where it may be a matter of considerable probability that organic disease may already be about to commence its dangerous operations." But this description embraces about nine-tenths of the cases of amenorrhœa daily presenting themselves for treatment! How rarely does a physician see any but a chronic case of amenorrhœa? How seldom does he meet it, whether in the relation of cause or effect, without disturbance of the different actions of the body? Above all, if organic disease be present or apprehended,—and we must suppose Dr. Davis to include cancer equally with other organic diseases,—is mercury, a medicine notoriously producing mischievous effects in cancer, one that he would seriously recommend our having recourse to?

We cannot, therefore, avoid raising our voice against the empirical practice here inculcated, as attended with the grave and serious objections of being an unnecessary, painful, hurtful, and possibly even a fatal method of treatment, in cases, the great proportion of which are simply and easily managed, upon ordinary and well-understood pathological principles.

With the exception of his unsparing exhibition of mercury in its treatment,—and to which the strictures just made in the case of amenorrhœa are equally applicable,—we look upon that part of our author's work which treats of Leucorrhœa as generally practical and instructive; but more particularly that portion of it which refers to the production of leucorrhœa as the effect of pregnancy and mismanagement in labour. To this we would particularly request our readers' attention. Dr. Davis seems to place much confidence in tincture of cantharides in this affection; and, although he does not go to the same full doses as Dewees, he recommends pushing the medicine sufficiently far to secure its specific effects on the genito-urinary organs. He speaks of fifty drops three times a day as his maximum dose; and this we esteem safer than the plan recommended by its great advocate, Roberton, whose recorded cases exhibit such a reckless perseverance in this painful method of treatment. We do not think our author devotes the attention merited to local treatment in this disease; he makes no allusion to Dr. Jewel's plan of treatment, which has acquired so much the confidence of the profession, and only mentions nitrate of silver in a cursory, general allusion to vaginal lotions.

Our author is opposed, to a certain extent, to the explanation offered, and the practice adopted, in the disease so well described by Dr. Gooch as the Irritable Uterus. He contends that this disease is, if not actually inflammatory, at least one of no inconsiderable vascular congestion; and that there is, as well as a morbid state of the nerves of the affected organ, a morbid over-distention of its blood-vessels. He consequently considers local depletion as constituting the most efficient part of the practice: for this purpose he applies leeches to the uterus itself. He has not experienced benefit from the use of iron, unless when the irritation depended upon leucorrhœa or painful menstruation; and in the former he prefers ample doses of the carbonate. The warm bath he objects to, from the inconveniences produced by change of posture; being quite as decided an advocate for the protracted and constant retention of the recumbent position as Dr. Gooch. The above means, with mercury to the production of salivation, (the author's sovereign remedy in all cases of difficulty,) he has principally relied upon; having, as he states, employed aperients, narcotics, and other classes of medicine, more as auxiliary than as principal remedies.

Under the head of Peculiar Diseases of Women, variously classed by nosologists, our author treats of Hysteria, Nymphomania, and Sterility. His observations on the first of these subjects are replete with learning, and evince much research on the part of the writer. Although he is at much pains to exhibit the striking connexion existing between this remarkable disease and uterine derangement or disease, as urged by Pinel, Villermay, Pujol, and others, yet he is himself of opinion that the hypothesis of its origin being in some morbid but unknown condition of the cerebral system, may deserve to be considered the most probable. He devotes much of his materials to prove and elucidate the striking connexion between the feelings and passions (but more especially the all-absorbing one, love), and this disease; and his observations, although to some they may appear diffuse, and even not very pertinent to the discussion of a medical question, seem to us highly interesting, and convey very useful hints to the practitioner in the management of cases most perplexing and of very common occurrence; cases in which moral management, and the exercise of acuteness and judgment on the part of the professional attendant, are often much more important than any treatment of a mere medicinal kind. As a means of effecting an alleviation of the spasms observable in hysteria, Dr. Davis recommends friction, which may be applied with considerable force to the part affected. In this way, rubbing the throat in the globus, as well as the scrobiculus cordis and abdomen, in those cases where spasm of the diaphragm, abdominal muscles, or muscular tract of the intestines, proves distressing, has, according to his experience, produced decided relief.

In proceeding to the general treatment of hysteria, the author seems hardly to act in perfect consistence with his own views of its essential nature; or, at least, to attend to the collateral phenomena in the first place. Being of opinion that few patients menstruate with perfect regularity, and, moreover, that, in the great majority of cases, it originates in some imperfection or disturbance in the performance of the catamenial function; and that embarrassments of the circulation are almost always the very speedy consequence of suppressed menstruation, he recommends the practitioner's first attention to be directed to the state of the circulation. When found embarrassed, or much disturbed and irregular as to ts determinations, the most vigorous means for its restoration to its natural and healthy balance must be adopted. There are two great measures, which, if used opportunely, are, according to Dr. Davis, especially calculated to restore the circulation (when disturbed, as here supposed,) to its natural and healthy balance: these are, first, venesection as soon as possible after the application of the cause of suspension of the catamenial function; and, secondly, when necessary, the exhibition of "a moderate charge" of mercury: one sufficient, however, to produce an ascertainable affection of the mouth. For our own part, although we confess we never have been subject to the general dread which prevails in the profession with regard to the abstraction of blood in hysteric patients, where symptoms were such as to require this treatment, yet we feel quite satisfied that Dr. Davis will not be likely to make many converts to his plan of practice amongst British practitioners. We certainly think that he has not done sufficient justice to the class of individuals to which these hysterical patients belong, and how little they appear to promise from a depletory plan of treatment; to say nothing of the likelihood of the benefit to be derived from a course of mercury where, judging from our ordinarily accepted principles of disease, every thing would seem to contraindicate it. This we certainly must observe, that in a recent case, as mentioned by Dr. Davis, unless it were very unmanageable indeed, we would esteem the having recourse to so violent and disagreeable a plan of treatment as mercurialization as unjustifiable and uncalled for; having at our command ample means of controlling or checking the disease on much better terms for our patient. We confess we felt rather disappointed at Dr. Davis, who exhibits so much early research in this article, not bringing his information down to the present day: we find no notice taken of the discoveries in spinal irritation, as connected with hysteria, beyond two lines quoted from authors, both of this and other countries, who have laboured to give a prominence, both in the pathology and practice, to the essential dependence of the disease on disorder of the nervous centres.

We have already remarked upon the inequality shown in apportioning the materials of this book; we are again struck with this fact in observing the unnecessary length of the article on Nymphomania; a disease which our author himself considers as very rare, and the enquiry into which he pronounces to be more curious than profitable. This strikes us the more forcibly when we contrast it with the article on that most important and frequent disease, dysmenorrhœa, which occupies not two pages, whilst this, on nymphomania, is extended to upwards of forty!

A contrast is drawn between Erotomania, in which there is an entire absence of sexual importunities, and that form of mania more especially characterized as nymphomania. The first is simply intense, all-absorbing love; and often, at least in the first instance, a monomania, although it is liable to terminate in the latter or more deplorable form. Dr. Davis is at great pains to describe most vividly these different affections, and to connect them, as far as he is warranted by pathological investigation, with physical disease. The latter form, nymphomania, has, he says, been generally found confined, either in the relation of cause or effect, with inflammatory or organic disease of the genital organs, but more especially the uterus, ovaries, and clitoris; an opinion with which we cannot coincide, as far at least as the ovaries are concerned, after the statement made by us in the review of Dr. Löwenhardt's work on Oophoritis, in our last volume.

In his observations on Tuberculated or Membrano-fibrous Tumours of the Uterus, our author reports an interesting case, in which he attempted to remove a diseased structure which was contained more within the cavity than in the parietes of the organ. Although his attempt failed, yet the hint is a useful one, and it appears to have been attended with the remarkable benefit of checking the hemorrhage, which had been distressing. The stoppage of the hemorrhage is explained by the cohesion and consolidation of the surface of the tumour with that of the uterus at the part where the separation was affected; the loose membrano-vascular tissue previously connecting which, he looked upon as the seat of the hemorrhage. We were, however, much surprised at finding no notice whatever taken by Dr. Davis of Wenzel's interesting observations and beautiful delineations of these organic uterine structures: indeed, the author seems to be ignorant of the peculiarity of its attacking so markedly certain parts of the uterus, and confining its development to them; a fact Wenzel has so well explained and exhibited.

Where such men as Hunter and Denman have included an inverted uterus in their ligatures, for the removal of polypus, we can properly estimate the caution necessary in this operation. The plan of loosening the ligature, and even removing it when symptoms of an alarming nature occur, as practised and recommended by Herbiniaux, Denman, and others, is very judiciously insisted upon by our author. He does not, however, seem to be aware of the views of Siebold and other continental practitioners as to the absence of vascularity of polypus; or, if he be, he is evidently not a convert to their doctrines, from the plan he prefers of operating upon these cases. He avows himself a supporter of the French plan of applying the ligature merely as a tourniquet, for the prevention of hemorrhage, and removes the tumour with the knife. As we made some observations on this subject in our last Number, on reviewing Dr. Hamilton's work, we shall only further remark, that our author's observations on this subject generally, but more especially in the department of diagnosis, are highly practical and instructive. Gooch's double canula he seems generally to prefer, although he speaks favorably of Desault's instrument in certain cases of very large polypus.

There is scarcely any morbid growth of the female genital organs that has not been at various times classed under the head of Moles. We esteem the definition of Fernelius as the most accurate and comprehensive: "Mola est tumor carnosus non in substantia, sed uteri capacitate genitus."* The early writers on this subject treated of moles of *generation* and *nutrition* under distinct heads. The prevailing opinion, however, appears to be in favour of their occurrence as the effect of sexual congress, or rather misconception. We perceive Dr. Davis is of opinion that this class of morbid growths may occur in the virgin, although rarely. He does not seem to have consulted Lamzweerde's celebrated "Historia Molarum," else he could scarcely have supported so heterodox an opinion. So satisfied was this author as to moles being the effect of vitiated seminal deposit, that, in order to relieve himself from his difficulties of asserted malformation in the virgin, he seriously devotes a chapter to discuss, *An virgo vel vidua ex diabolo possit concipere molam?*

Of the necessity for attending to the diagnosis in these cases, and their resemblance to pregnancy, we may form some idea, when we have on record cases in which the wives of the most eminent medical men have proved the subject of blunders of this kind. We must, therefore, see the propriety and importance of correct diagnosis on this subject; and we confess, under this head, as well as in the case of hydatids generally, Dr. Davis disappoints us. The rules he attempts to lay down in both are unsatisfactory and inconclusive; and the best means of diagnosticating between these affections and pregnancy,—namely, auscultation,—in the one instance he omits entirely all mention of, and in the other slightingly and unjustifiably pronounces to be inapplicable.

The author, commenting on the operation suggested by Dr. Marshall Hall, and practised, with different modifications, by foreign surgeons, for the purpose of affording support to the uterus in cases of prolapsus or procidentia of this organ, proposes another operation instead, by which the possibility of any inconvenience from hemorrhage will be obviated, and a sufficient amount of inflammation excited to ensure the readhesion of the parietes of the vagina to their naturally contiguous tissues within the pelvis. Instead of a continuous incision, or incisions through the lateral parietes of the vagina, his idea is that of "passing through the same lateral parietes of the vagina a series or cluster of acupuncturations sufficiently far into the cellular membrane to ensure the effect wished to be obtained from the operation." For this purpose, the author recommends an instrument analogous to that used for cupping, armed with pins or needles, instead of lancets, calculated to make punctures, which should be carried directly through and deeply into the cellular membrane beyond the lateral parietes of the vagina. There is some ingenuity in this suggestion; but it wants, what the other methods possess, the sanction of experience.

The following are the author's directions for the reduction of Ante-

* Lib. vii. Path. cap. 15.

version of the Uterus. After premising leeching, cataplasms, rest, &c., he proceeds:

"The patient should be made to lie on her back, with her breech elevated above the level of the upper part of her trunk, so, as much as possible, to relieve the uterus from the weight of the intestines. The bladder and rectum being previously well emptied, the index and long finger of the left hand, *if long enough*, should be passed high up into the vagina, and beyond the vaginal part of the uterus, where they should be made to exert an effort to dislodge that part of the organ from its preternatural position, by applying considerable force to it from above and behind downwards. If the fingers cannot be made to reach the part of the uterus in question, then a slightly curved instrument, of sufficient length, made of wood, and rounded smoothly into an extremity not unlike that of a common vectis, must be substituted for them. Whilst the effect here supposed is attempted to be produced on the vaginal part of the uterus, another instrument, curved or not, as the case might indicate, but broadly padded at its extremity, should be introduced immediately behind the symphysis pubes, and so applied to the tumour formed by the fundus and body of the uterus as, by a firm bearing upwards of its padded extremity, should effect a perceptible change of position of these parts; or, in other words, such a bearing upwards, acting in combination with the counter-pressure downwards, made, as already directed, on the vaginal portion of the uterus by the other instrument, as should suffice to effect the reduction of the entire organ."

We would make the same remark on this mode of operating which we made on Dr. Davis's mode of measuring the diameter of the pelvis:—it is too elaborate, too instrumental, too harsh.

We shall not dwell on the article on Malignant Diseases of the Uterus; it is too much occupied with discussions and quotations from other authors on the supposed texture of the disease, to the neglect of remedial or alleviative treatment.—The chapter on Diseases of the Lateral Appendages of the Uterus is scanty, when compared with the general character of the work.—Our author adopts Sir A. Cooper's arrangement of Mammary Diseases, from whom he judiciously quotes so largely as to render this article nearly a reprint of Sir Astley's very valuable work.

We come now to Pregnancy, and its consequences. Dr. Davis considers Blumenbach's views of generation the most rational, and adheres to Hunter's theory with regard to the placenta. But, to our astonishment, we observe that he omits all mention of Baer, and Prevost, and Dumas's important and most interesting experiments and observations.

While reviewing a work which displays so much laboured research, we cannot omit expressing our regret that the section on the Signs and Indications of Pregnancy is so incomplete. The author, speaking of the change of colour and size of the areola round the nipple, says, (p. 854,) "Some people, therefore, lay much stress upon it as an indication of pregnancy; and it may be the *best single evidence* which we possess." Further on he says, however, "In a small proportion of women it may afford little or no assistance; as in females of very fair complexion, in some of whom no perceptible change of colour takes place, either during pregnancy or even during the puerperal state; whereas, in other subjects, the areola has presented a deepish tinge in the absence of pregnancy, and even in persons who had not been pregnant at any former period. Nursing has always the effect of keeping up the appearance of it." We feel quite satisfied that too much confidence has been reposed in this evidence of pregnancy; nor are we willing to attach any more importance to it than as a single symptom, which, as a guide to diagnosis, is very far indeed from being infallible, but which, in conjunction with others, affords some assistance in forming our opinion as to the nature of the case. The excellent observations of Dr. Montgomery, in the Cyclopædia of Practical Medicine, are the best yet presented to the profession on this most important subject.

Quickening is referred by the author, with Dr. Seguin Jackson, to the rising of the uterus out of the pelvis, and entrance into the abdominal cavity. Dr. Davis has omitted all mention of Abattement, Ballottement, and even Auscultation! The omission of this latter as one of the means of detecting pregnancy, in a work published at the present day, we cannot account for. Surely, although the author may not have been aware of the more recent labours in this department of medical science by Dr. Evory Kennedy in his own country, Dubois in France, and Hohl and Kilian in Germany, he can hardly have been unacquainted with those of Major, Kergaradec, and Laennec, now many years before the public. We can, from considerable experience, assert it to be infinitely the most satisfactory and conclusive means of diagnosis we possess. We regard this as one great blemish of Dr. Davis's work; and we feel it therefore necessary, in justification of British obstetrical medicine, to state that Dr. Davis's view of this matter constitutes the exception, not the rule, of practice among our best accoucheurs. We take this opportunity of recommending to all not already acquainted with it, the excellent treatise of Dr. Kennedy on this subject.

In describing the operation for reducing the Retroverted Uterus, the author recommends (we think judiciously,) when, "after a time, the fingers may be conducted so high as to be found not long enough to reach the fundus uteri, the passing up of a piece of cane of considerable thickness, with a broad, firm, and finely textured piece of sponge well secured to its top."

In the treatment for Œdema of the Lower Extremities from Pressure, the recumbent position, or keeping the extremities elevated as much as possible, is not mentioned. We doubt how far, where erysipelas is threatening, scarifications, though moderate, will tend to arrest it.

The author reports a very interesting case, as one of a number of important complications of diseased action with pregnancy, at page 887. It is a case of diarrhœa, occurring monthly during three successive pregnancies; it continued on each occasion for seven or eight days, with from fourteen to five-and-twenty copious alvine discharges each day. This case is again recorded (we suppose from oversight,) at page 901, as an instance of the occurrence of some unusual symptoms which have been recognized as signs of pregnancy.

The chapter on Extra-uterine Fœtation displays much research, and is well worthy of an attentive perusal.

In treating of Parturition, the author classifies labours into natural preternatural, complex, and instrumental. He excludes the consideration of time, and bases his arrangement, in the first two, on the nature of the presenting part; in the third, on "the occurrence of any occasional circumstance calculated to embarrass the function of parturition, and compromise its results;" and in the fourth, on the necessity of obstetric instruments for delivery. We shall not quarrel with this arrangement, but proceed to make a few remarks on the practice enjoined. Dr. Davis advises, when the head comes to distend the perineum, instead of applying the palm of the hand to the perineal tumour, to oppose the whole of our modifying and resisting force to the presenting part of the fœtal head, by the application of the points of the fingers and thumb of the right hand. This is a practice not only useful but essentially necessary, as laid down by Denman, in cases where the labour-pains are violent; but we can hardly suppose our author to mean that support should not at the same time be afforded directly to the perineum itself: if such be his doctrine, we must certainly record our dissent. The further directions, with regard to the expulsion of the child, are good; save that he omits the directions given by Dr. Clarke, of Dublin, "of pursuing, with a hand on the abdomen, the fundus uteri in its contraction, until the fœtus be entirely expelled, and afterwards continuing for some time this pressure, to keep it, if possible, in a contracted state;" the utility of which practice is so ably and satisfactorily demonstrated by Dr. Collins, of Dublin, in his Practical Treatise lately noticed in this Review. We cannot agree with Dr. Davis in his opinion as to the "use of the binder in ordinary cases being very limited," and "in many cases rather questionable;" more especially after the directions he lays down as to the removal of the placenta "in ordinary cases;" calculated, as we consider them, to encourage impatience, and, as a necessary consequence, lead to a meddling interference.

In labour protracted from rigidity, our author shows himself a true admirer of the doctrines of free depletion, and seems inclined to outrival Dewees in the extent to which he carries this practice. We would recommend him to enquire into the plan of treatment adopted by our Dublin brethren in such cases, where nauseating doses of tartar emetic have been found to effect the desired object; thus superseding the necessity for such copious and debilitating bleedings as he and his transatlantic friends advise.

Cases of deficient activity or inertness of the uterus are, the author states, especially to be treated by ergoted rye, in half-drachm doses every ten minutes, till the required effect be produced. This we consider too sweeping a decision, having known, in many instances, the life of the child to pay the forfeit of such indiscriminate practice. We have frequently, in cases under the full action of the ergot, administered even in scruple doses, heard the fœtal pulsation lowered from 130 to 50 in the minute, and eventually it has ceased altogether. We are surprised that Dr. Davis has omitted all mention of the benefit, in such cases, derived from the occasional administration of a stimulating enema. Its action may not always be so rapid, but it is surely far more safe.

The distinction drawn between impaction and arrest of the head is concise and good; and our author's observations on the circumstances most deserving of the practitioner's attention during his attendance on cases of protracted labours are sound, and well entitled to a careful perusal: were we to particularize, we would select those on retention of urine.

In breech presentation, where the back of the fœtus is directed towards the back of the mother, the author turns the breech round in the pelvis; and if, from disproportion, this cannot be effected by the fingers alone, he recommends the use of the blunt hook. We would say, do not try to turn: the body will always turn of itself before the head enters the brim, if not interfered with. Dr. Collins states (not alluding to the above practice, but the occasional necessity for instrumental delivery in breech presentations,) that, out of between 24,000 and 25,000 deliveries, but one instance was met with where any instrument was required.

We cannot possibly pass unnoticed Dr. Davis's observations on Twin Births. His introduction of this subject is confused, and calculated to mislead the student; as he commences by laying down rules for the management of those rare cases in which the children become engaged together in the pelvis, in place of impressing upon the mind of his reader the general rules applicable to all twin births. Whilst he very properly recommends the application of a bandage and keeping a moderately firm pressure upon the abdomen during the second labour, instead of directing the rupture of the membranes of the second child as the invariable practice, and most certain means of ensuring the contraction of the uterus, he merely casually alludes to this as what "*might* be usually required;" and this after a previous disquisition on the utility of bleeding, ergot of rye, and *the use of the forceps.* Upon this latter practice, however, the use of the forceps, we esteem his observations as more than objectionable,—we had almost said censurable: let our readers judge from his own words.

"If," he says, "the subject of the case shall have had children previously, the birth of a first child of a suspected case of twins might often be quickened and completed by having recourse to the forceps; and cases of this kind might be *made available by a young practitioner, for accustoming himself to the use of that instrument;* which, however, he will, of course, be careful to use with proper attention to the precautions to be hereafter detailed on the subject of instrumental midwifery." (P. 1019.)

Such a recommendation as this, coming upon the authority of the professor of midwifery to the London University, we cannot but regard as calculated to produce very serious mischief.

In Labours complicated with Prolapsus of the Umbilical Cord, Dr. Davis recommends turning, or, where this cannot be effected, carrying up the prolapsed portion by means of a thin flexible spatula. We recommend to our readers the observations of Dr. Michaelis on this subject in a recent Number of this Journal.

The section on Convulsions is interesting; nine out of ten, the author says, recover. We rather think this will be found to be too favorable a report. Dr. Davis feels himself warranted by experience in recommending the exhibition of a full dose of opium *after* ample bleeding; and, should the patient not be in a situation to swallow it, an opiate enema of proportional strength. His mind is made up on this questio vexata. We could wish he had described the state of the patient where he would give a full opiate. That there will be cases benefited by such treatment we admit, but we cannot entirely assent to the general recommendation, even *after ample bleeding.*

In treating of abortion, the author does not allude to the advantage derived from injections by the rectum in cases where the secundines have been retained, nor the exhibition of ergot; he gives very judicious directions as to the treatment to be adopted, viz. warm water injections into the uterus, when the discharges are becoming foul. In unavoidable Hemorrhage, where the discharge is considerable and the os uteri not disposed to yield sufficiently to admit of the introduction of the hand, Dr. Davis recommends rupturing the membranes as a means of "suspending uterine hemorrhage," and also of "inducing the action of parturition." That the evacuation of the liquor amnii is frequently followed by an arrest of accidental hemorrhage is well known, but it is not so certain that hemorrhage from an unavoidable cause will be thus checked. The discharge in this latter case is observed to be more profuse during uterine action, and this for obvious reasons, owing to the dilatation of the os uteri, consequent on this uterine action, laying open the mouths of the bleeding vessels: where the membranes have been ruptured, the hemorrhage can alone be stayed by the head, or some other resisting portion of the fœtus being firmly pressed against the gaping mouths of the vessels. But in few, if any cases, does the *os uteri continue unyielding so long as to endanger the life of the patient from the repeated losses of blood;* and should not the evacuation of the liquor amnii, on his own showing, be followed by an arrest of the discharge, the subsequent delivery will be attended with much more difficulty to the operator, and increased risk to the patient. We cannot therefore lend our assent to the practice recommended by our author.

In the remarks on hemorrhages after the expulsion of the child, Dr. Davis directs, judiciously enough, the removal of the placenta, but advises it, as we think, in such a careless way as to call for some observation. Surely it is not enough to remove the contents of the uterus under such circumstances; it is equally essential to effect the complete contraction of the uterus. For this purpose he relies on the uterine tourniquet, without alluding to what, in the cases under consideration, viz. hemorrhage from want of uterine action, is found the best mode of ensuring safety to the patient, stimulating, before withdrawing the hand, the uterus, so as to make it by its own action expel both placenta and hand. The uterine tourniquet will be, we have no doubt, a good mode of applying pressure, but we cannot approve of the practice of plugging the vagina after the removal of the placenta, even with the seeming guarantee against the possibility of subsequent relaxation afforded by the uterine tourniquet. We have found, in cases of such tendency to relaxation, the best effects follow on the administration of the ergot of rye; we are surprised Dr. Davis, who recommends it so strongly in labours protracted from inertness of the uterus, and is so satisfied of its power of exciting indolent uterine action, should have overlooked its usefulness under these nearly similar circumstances. In retention of the placenta from hour-glass contraction, where there is no hemorrhage, the author trusts to time (two or three hours may be required) or the exhibition of an opiate, which he considers sufficient: when hemorrhage is present, he at once introduces the hand and effects its removal. We have to regret that he has omitted to notice the difficulty attendant on passing the hand in these cases, as also the precaution to be used to ensure a final regular contraction; indeed, this section is rather scanty. We feel disappointed at his hardly alluding to the occurrence of internal hemorrhage, and the means found useful for its suppression. He states "that he has little to say on the different modes made use of to excite contraction of the uterus by irritating its internal surface;" and again, "moreover the author has seldom had recourse to the introduction of the hand for the purpose of exciting irritation. But in those cases where he has adopted that course, he has indeed sometimes succeeded; although at other times, and perhaps equally frequently, he has to regret that he has been greatly disappointed as to the result." We certainly were not prepared for this statement, nor does it at all tally with our experience. His plan of treating morbid adhesions is judicious.

Although it cannot be expected that we should go at much length into the portion of the work treating of Instrumental Delivery, as the substance of it has been for upwards of ten years before the public, this being little more than a reprint of Dr. Davis's Elements of Practical Midwifery, yet, as it forms a part of his system, we cannot leave it entirely unnoticed. Our remarks shall however be very few. It is not our intention to follow our author through his discussion on the different kinds of forceps, or mode of application: on these points we refer our readers to the work itself; but we cannot, however, help thinking, from the familiar and easy manner in which he speaks of withdrawing, changing, and reintroducing the blades of the forceps, he seems to have lost sight of the fact that one of the principal risks attendant on the use of this instrument exists in their introduction, and that even the withdrawal of a blade when the child is firmly pressed against the soft parts and pelvis, is not unattended with serious danger of lesion. We must enter our protest against the following practice:

"If we suppose," (says Dr. Davis,) "the forceps to be often used as a mere auxiliary to assist in overcoming local or temporary difficulties, or in cautiously effecting the dilatation of rigid parts, and not systematically to be kept in application until the delivery of the whole head is accomplished; it is evident that such a limited use of them may be had recourse to with much greater frequence than if we suppose a rigid adherence to the opposite but very common practice." (P. 1142.)

Such a practice, sanctioned by so high an authority, we cannot but consider likely to encourage a too ready resort to the use of instruments. As to the plan recommended of protecting the soft parts of the mother by securing a flannel pad or cushion to the convexity of the forceps, and thus adding of course to their bulk, we freely confess we have never tried it, conceiving that such a proceeding would increase the difficulty in their introduction; being also of opinion that cases which would admit of such a mode of delivery, would, by a little exercise of patience, be brought to a happy termination by the unaided efforts of nature. The author has omitted, in his otherwise accurate details, to dwell upon the signs of the death of the fœtus in utero. This we consider of much importance, inasmuch as in many instances it might have some weight in determining the instruments we would select for delivery. Auscultation, again hardly alluded to by our author, will be found most valuable in deciding this point.

From the circumstance already alluded to, with regard to the previous publication of the part devoted to instrumental delivery, we have directed our attention principally to the matter contained in the other divisions of these volumes; and we must now draw all our remarks to a close. The observations on the Diseases of Children in the third part, which are comparatively so concise, occupying only about sixty pages, we can no further advert to than to state that the subjects there treated of are for the most part discussed practically, although restrictedly.

We must here terminate our notice of this work. Our opinion of its actual and relative importance may be inferred from the pains we have taken to enable us to form a just opinion of it, and to deliver this honestly and explicitly to our readers. The first part of Dr. Davis's treatise, or at least the greater proportion of it, we esteem comprehensive beyond any work in the English language. It evidences great labour and unwearied research. The references and cases contained in it render it an invaluable addition to the obstetric library. In its present form, however, it is more likely to be used as a book of reference by the more advanced practitioner, than a class-book by the student. Not that we by any means recommend or sanction the too prevalent reading-made-easy system of primer instruction: on the contrary, in our opinion, it strikes at the root of science, and pushes artificially into practice a class of ill informed, injudicious, and consequently often mischievous individuals, who have but one rule of practice to adopt under the protean varieties diseases assume; and who are only, at the termination of a tolerably long life, beginning to learn, what observation at the bedside, and an intimate acquaintance with a sufficient number of well selected authors, should have long since taught them.

Although a work of great research, the reader will not fail to have remarked, that it is decidedly defective with regard to some of the most marked improvements which have taken place in this department of medical science within the last few years. To this indeed it may be objected that the work has been coming out in parts; but, the improvements, to which we allude, have almost all been known previous to the appearance of the first part. Even had this not been the case, it would not have been a very difficult matter to have introduced them in an appendix. We must, however, in candour admit that, though we have found not a little to condemn, still the greater part of the work has earned our unqualified approbation, as containing not only sound and practical, but also original matter.

By some it may be considered that we have been unnecessarily severe in our strictures; and we freely confess that a writer in a less exalted situation and coming before the public with fewer pretensions, might have been less decidedly censured, while he would have been less critically noticed. The case, however, is different with our author; his station almost ensures his work being received as an authority throughout this country, and by foreigners as the representative of British doctrines; we felt, therefore, conscientiously compelled to denounce whatever practice and experience satisfied us was objectionable in the statements put forward; we trust we shall not be considered to have done any thing more.

II. In entering on a review of the work of Dr. KILIAN, we have, at the very outset, to complain of much useless verbosity, and also of a scholastic pomposity, which, from its universal prevalence, is exceedingly annoying to the reader. In writing on subjects connected with Midwifery, or any other branch of medicine, every thing like gaudy description or floridness of style should be carefully avoided: it should be concise but comprehensive, simple but dignified: never, perhaps, were these qualities so remarkably combined as in the works of the celebrated Richter.

In Chapter I., speaking of examination with the hand through the abdominal parietes, Professor Kilian mentions the names of Rœderer and W. J. Schmitt; but he neglects to inform his reader in what particular respect are these names connected with the above subject. The chief peculiarity in their mode of examination was, that, having placed the patient in such a posture as should effectually relax the abdominal parietes, by raising the knees and shoulders, they directed her to breathe slowly and deeply, and, during expiration, made pressure with the hand upon the abdomen.

The author, in his directions for examining per rectum, (p. 96,) informs us that "the finger should be *rapidly* introduced, with a twisting motion, through the orificium ani. The operator here must be quick, because, if he attempts to introduce the finger slowly, it may happen that the sphincters contract firmly, and close the intestine." We have always pursued a diametrically opposite treatment, insinuating the finger as slowly and gently as possible; and feel assured that, from the irritability of the sphincter, it would be quite impossible to pass in the finger suddenly without using an improper degree of force, and, at all events, putting the patient to much suffering.

The second Chapter is devoted to the Obstetric Measurement of the Pelvis. The author commences with a flourishing assertion, which we quote literally, in order to afford our readers a specimen of his verbose and pedantic style.

"It doubtless belongs to the most remarkable, aye, wonderful and unheard-of facts in our profession, that accoucheurs of every century, till the time of Deventer, had in no wise considered the pelvis and its defects, as causes of difficult labour; yea, that they had never once thought of it as a possible obstruction of labour, just as if there had been no pelvis at all." (P. 102.)

Does the Professor forget Mauriceau? a man who, considering the state in which he found it, contributed more to advance midwifery than any practitioner before or since his time; not even excepting our own celebrated Smellie. We do not see the use of enumerating all the various pelvimeters which have been invented, because, with the exception of Baudelocque's *compas d'épaisseur,* they have long since been proved to be useless, and are therefore deservedly forgotten. Indeed, we may say that the last paragraph of the chapter contains the sum total of the previous thirty-one pages; viz. that the hand of an experienced accoucheur is, after all, the best instrument for ascertaining, not only the size and shape, but also the condition of the pelvis.

Professor Kilian's third Chapter is entitled "on the Operative Treatment during the fourth and fifth Stages of Labour," and is divided into five sections: the first relates to the species of bed which the patient must use, and the position which she is to maintain during labour. In speaking of labour-chairs, he omits the name of Ambrose Paré, one of its earlier inventors. Again, in speaking of the labour-cushion, he merely mentions the names of Siebold and Carus, entirely omitting that of Joh. Unger, who claims a much greater priority. The Professor does not exactly approve of our English method of delivering a woman on her left side, although it is used in Germany by some of the most distinguished practitioners. Here, unfortunately, he is again at fault with his literature, and omits the name of Fielding Ould, who strongly recommends this position.

The author's observations on supporting the perineum, which forms the subject of the second section, are not more successful. The sum of the whole is, "that rupture of the perineum occurs almost as frequently when it is supported as when it is not; except that, when it is supported, the rupture is not so extensive. Michaelis's proposition (to *cut* the perineum,) is of no value; the perineum must be supported by the hand, protected by a napkin, and this support should be continued until the shoulders are born. A proper conduct on the part of the patient at this moment is as important as the support of the practitioner."

Madame la Chapelle's observations on the effects of pressing the perineum where the pains are slow ought to have been mentioned, where such a display is made of acquaintance with the literature of the subject. We ourselves have no experience as to the efficacy of such treatment.

The directions for receiving the child (sect. 3,) contain nothing remarkable. In tying the cord, the author informs us that the English accoucheurs of the present day tie the cord *immediately* after the birth of the child: this we must take the liberty of contradicting. His directions for instantly tying the cord where the child does not breathe, and not to attempt resuscitation whilst the connexion between the child and placenta remains, are faulty and mischievous in the extreme. The scissors ought *not* to be sharp, but rather blunt, in order that, by contusing the coats of the umbilical vessels, a greater degree of contraction may be produced, and thus the danger of hemorrhage diminished, in case the ligature slips or becomes loose.

Section 4, on Delivering the Placenta, contains nothing of interest. Much space is wasted in dilating upon the old method of delivering the placenta *immediately* after the birth of the child. In delivering a twin-placenta, the author directs us to pull first at one, then at the other cord. A bad mode of practice; as we are thus liable to tear the cord from its insertion, and separate the placenta unequally. We should twist the cords together; the strain is thus divided more equally, there is less danger of their rupturing, and the placentæ come away better.

Chapter IV. treats on the Artificial Dilatation of the Os Uteri. This is divided into two sections: the first, where it is effected by mechanical stretching; the second, where cutting instruments are employed.

The historical introduction shows that the specula for dilating the os uteri are now no longer used; the hand being the only instrument for that purpose. The indications for the operation are very incorrectly stated; nor do we think they would have been at all improved if Prof. Ritgen had even added twenty more indications to the twenty he has already given; they only tend to prove that neither he nor Prof. Kilian have any clear notion upon the subject. Dr. Feist, in his review of the Professor's work, has mentioned four indications: "First, violent, dangerous, and uncontrollable hemorrhages, (especially in placenta prævia;) secondly, severe convulsions; thirdly, apoplexy; fourthly, retained placenta, with contracted os uteri, which cannot be removed by any other means." To these indications we also object. According to our own opinion,—and it is based on high authority,—we should say that the artificial dilatation of the os uteri is *never* indicated before the birth of the child: that we occasionally meet with cases where the introduction of our hand is justifiable before the os uteri has quite attained its full degree of dilatation, is well known; but this is very different from the artificial dilatation of it. We should say, that in *no* case of hemorrhage is it indicated; because, where the patient is so seriously exhausted that we dare not wait to plug the vagina, the os uteri will be found quite relaxed, and capable of admitting the hand. The placenta being situated upon the os uteri ought rather to be a *contra-indication* to artificially dilating: our reasons for saying so we have already stated, in our review of Dr. Collins's work, (No. III. p. 83.)

In Rigby's Essay on Uterine Hemorrhage, we find that he expressly cautions us "against the premature introduction of the hand, and the too forcible dilatation of the os uteri, before it is sufficiently relaxed by pain or discharge, &c." His observations which follow show that this opinion was not the result of theory, but of experience. We deny also that artificial dilatation of the os uteri is indicated, or even justifiable, in severe puerperal convulsions: we should only run the risk of aggravating the disease to a fatal degree. That delivery of the child under such circumstances is most desirable, we do not deny. "The patient," says Dr. Burns, "should be delivered as soon as we can possibly do it *without violence*." Dr. Feist's third indication is in great measure liable to the same objections; the artificial dilatation of the os uteri being only justifiable in cases of apoplexy, where it has already become relaxed by bleeding, uterine contractions, and other means. It is only where the os uteri is contracted upon the placenta after the birth of the child, that we consider artificial dilatation of it to be indicated; and here the directions so correctly and simply laid down by Celsus are most fully applicable.

Professor Kilian, however, is not satisfied with all this; but feels convinced that the other operation is in many cases infinitely preferable; or, in other words, he recommends nothing less than cutting open the os uteri with a knife in some of the above-mentioned conditions, as acting more rapidly than by dilatation with the hand. In speaking of this favorite operation he gives four indications, two only of which we can admit; viz. first, where the os uteri is partially or completely closed by adhesion; and, second, where it is rigid from callous cicatrices and scirrhous indurations. The sudden death of the mother at the commencement of labour is no indication for this operation, the os uteri being *spasmodically* contracted round the child, even if the cord be implicated, would not justify our dilating it with the knife. If the Professor could have contented himself with the admirable observations of Richter on this subject, as also on imperforate and contracted vagina, which ought to have been noticed, this chapter would have contained valuable matter, expressed in intelligible language.

Chapter V. is on the subject of Rupturing the Membranes, or, as the Professor latinizes, "Operatio ad rumpendas *aquas*." Professor Kilian considers that the old view of the os uteri being dilated by the pressure of the elastic cone formed by the membranes distended with liquor amnii is incorrect, and that the dilatation is "solely the result of a peculiar activity of the inferior segment of the uterus." With peculiar inconsistency he states, in a following page, that this extends equally over the whole uterus; and, a little further on, that it exists chiefly in the fundus. To this view, if it had been less confused, we should have had no particular objection; but we greatly object to the Professor appropriating the whole merit of the case to himself. Either he has never read Dr. Dewees's excellent observations on this subject, which are beyond all comparison better, or, if he has, that he has not thought fit (for reasons best known to himself,) to acknowledge it.

The indications for rupturing the membranes are, for the most part, incorrect, and would lead to bad practice: they betray a fondness for interfering with the regular process of a labour, which cannot be too strongly deprecated. We find the following observation at page 278, when speaking of the indications for rupturing the membranes:

"5thly. When we are desirous of fixing at the brim of the pelvis a presenting part, or a desirable part which has been made to present, and which we are uncertain will remain presenting, we not unfrequently see, in cases of spontaneous evolution (Selbstwendung), *where the membranes are unruptured,* that the part of the child which had presented favorably again recedes, and then again appears."

We do not complain of the indication, because cases do occur where it is desirable to rupture the membranes for this purpose; but what are we to understand by "Cases of spontaneous evolution where the membranes are unruptured?" This we cannot comprehend; at least, we never heard of such cases where the child had been carried the full time. Professor Kilian recommends rupturing the membranes to *diminish* the activity of the uterus, *where the contractions are too violent.* Most practitioners would adopt this practice with precisely the contrary intention.

Under the head of rupturing the membranes *before* labour, Professor Kilian brings on (and not very judiciously,) artificial premature labour. This forms the second section of the fifth chapter. The literature is given very minutely, and, but for the pompous diffuseness of the author's style, would have formed a very interesting article. He quotes the observation from Denman, that it was a practitioner named Macauley, in 1756, who first induced artificial premature labour, for the purpose of saving the child, and it is said with success. To Dr. Denman is due the merit of having first called the attention of the profession to the practical advantages of this operation. Weidmann first practised it in Germany, although the late Professor May first proposed it, in 1799, in his essay, "de necessitate partûs quandoque prematuri, &c. promovendi."

In his observations on the effects and utility of the operation, Professor Kilian combats the various objections which have been made against it, and shows the absurdity of some of the arguments which have been used for this purpose by the French practitioners.

Professor Kilian's observations on the size of the head during the last month of pregnancy contain nothing new; but he leaves an equally, if not more, important point unnoticed,—viz. the softness and yielding condition of its parietes at this period. It is not the *size* of the fœtal head which is alone to be considered in this operation, but the hardness and degree of ossification must be also taken into account. There is no excuse for this omission; the more so as he proceeds, in the most self-satisfied style, to mention several well-known facts, which we would suppose he intended us to consider as discoveries.

Professor Kilian expresses his conviction that the development of the fœtus may be retarded by deficient nourishment, depressing passions, diseases, &c. From this incorrect notion we, of course, strongly dissent. We know that, under the greatest deprivations of poverty, large and lusty children are born; that, in cases of seduction, &c., where pregnancy has been passed under the most overpowering despondency and wretchedness of mind, the unhappy mother has gone her full time, and has borne a fine robust infant. Nothing is more constantly observed than, in phthisis, where Nature appears to collect her last remaining powers, in order to complete the process of utero-gestation, and then sinks exhausted, that the child is generally healthy and strong, possessing every character and mark of full development. We are well aware that it was once proposed to retard the development of the fœtus, by bleeding, low diet, and other means of reducing the system; but the fallacy of this supposition has long since been proved; nor do we believe that it has ever been put into practice.

In speaking of the operation, a long and rather needless description is given of every modification of instrument which has been used for the purpose. He describes three ways in which the operation has been recommended to be performed: first, by puncturing the membranes; secondly, by artificially dilating the os uteri; and, thirdly, (Hamilton's plan,) by inducing uterine action, by separating the membranes to some extent from the uterus. He prefers opening the os uteri artificially, and then puncturing the membranes, a plan which cannot be recommended; and entirely omits mentioning the method recommended by May, and put in practice so successfully by Professor Naegelé,—viz. of first exciting uterine action by warm baths, a smart purge or two, abdominal frictions, and secale cornutum. The labour is thus made to resemble more completely a natural one: the membranes are not ruptured until the os uteri is dilated, and thus the child protected from the pressure of the uterus, which cannot but act prejudicially upon it where the liquor amnii has been drawn off prematurely.

We come now to the sixth and last chapter of the first volume,—viz. on Turning. This is divided into three sections: first, turning by bringing down the feet; second, by bringing down the nates; and thirdly, by bringing down the head.

The operation of turning has long been divided into two distinct acts; viz., first, artificially changing the position of the child; and secondly, the artificial delivery of it. Professor Kilian does not *distinctly* state this point, although he apparently intends to do so; and makes no mention of Aitken of Edinburgh, Foster of Dublin, or Wigand of Hamburgh, all of whom expressly noticed it. Stein, sen., as connected with the subject, ought also to have been noticed; and we find, from Dr. Feist's review, that the distinction "between *turning* and *extraction*" was clearly shown by the celebrated midwife, Justina Siegmund, so long ago as 1689; a circumstance we were not aware of. Dr. Feist has made an excellent remark on this subject, which we will beg leave to quote: "If the long axis of the child does not correspond with that of the uterus, turning becomes an *absolute* improvement of its position; but, if it presents with the head or nates, and cannot be delivered in this position, under urgent symptoms which require labour to be hastened, merely a *relative* improvement will be produced by bringing down the feet."

Professor Kilian's indications for turning we pass over: more directly against every fundamental principle of good practice in this branch of midwifery, they could not well have been. In describing the operation, one would imagine that he is endeavouring to select every objectionable point of practice possible. His first rule is, that we should know correctly the precise position of the child. This is very proper; but is it always possible? What would he do in placenta prævia? These same objections will apply to his rules for using the right or left hand. His rules as to what part of the pelvis the hand is to pass along are useless: it would have been infinitely better if he had followed Dr. Denman's excellent directions on this head; viz. to pass up the hand on that side where there is most room; but the name of Denman does not once occur here. His observations on the management of a contracted os uteri are vague and unconnected. Dr. Dewees's admirable remarks on this subject ought to have been mentioned, as also those of Wigand; but no notice is made of them. In introducing the hand into the uterus, the Professor informs us that the next point to be thought of is rupturing the membranes! We should say, that at this moment it ought to be our especial object *not* to rupture them; but M. Kilian reprobates the valuable practice laid down by Peu and Deleurye, of passing up the hand between the membranes and the uterus, until we reach the feet; and, from his reasons for so doing, evidently shows that he has no clear ideas upon the subject. Dr. Hamilton's remarks, also, on this important point are not noticed; and, on searching for the feet, no mention is made of Denman, although no one has given more simple and practical rules than he has on this point. The Professor (p. 405,) makes a good remark, as far as it goes, —viz. that, "as soon as the head has reached the fundus, and the hips the brim of the pelvis, the turning should be considered as completed." This is followed by no observation that the rest of the labour, now that the long axis of the child's body corresponds to that of the uterus, should be conducted according to the general rules for any other natural labour. His other directions, as to turning with the feet foremost, contain nothing peculiar, except that the method of turning the child upon its long axis, as proposed by Professor Deutsch, of Dorpat, has been mentioned. It consists of pushing up the shoulder by passing the hand under the axilla in such a direction that, whilst we raise the shoulder, we at the same time turn the back of the child towards the anterior parietes of the uterus, the feet will now be secured with the greatest ease. This operation is said to facilitate changing the position of the child under circumstances which would otherwise render it difficult: we have never attempted it in practice, and only know of its mode of action from having been shown it, some years ago, on the skeleton pelvis, by Dr. Deutsch, jun.

The Second Volume begins with rules for extracting with the feet foremost. The author appears to think that nature has nothing to do with this process, and does not appear to be aware of the important office which the fundus uteri performs, in pressing upon the head, in order to prevent the chin quitting the breast. He also hints that the accouchement force is necessary in placenta prævia!

The eighth Chapter is on the Forceps. To go into this interesting subject as fully as it deserves, would require us to dilate this article much beyond the limits we have assigned for it: we must therefore confine ourselves to the most important points, and notice them in as brief a manner as possible.

Forceps of a much better form, and with fenestræ, had already been used by Drinkwater, and *at least* as early as 1726, by Giffard; when nothing of the sort, beyond the clumsy unfenestrated blades of Palfyn, were known in Paris. They were improved to a certain extent, it is true, by Petit, &c.; but Chapman's forceps soon became those in common use at Paris, and were the origin of Gregoire's and other French forceps. Professor Kilian's history of the forceps is wretchedly meager and incomplete: here was a wide field for the display of extensive reading and literary acquirement, and it would have given us much pleasure to have seen this subject well handled. The improvement of the forceps, by adding the pelvic curvature, which was equally due to Levret and Smellie, is not mentioned; nor are the merits of their respective claims to the invention at all discussed. The author seems unacquainted with the short paper published in the Medical Gazette, several years ago, by Dr. Rigby.* The description of the Chamberlen instruments, which were found some years ago, and which was published by the same gentleman, ought to have been mentioned; but the Professor, as Dr. Feist observes, appears to have been ignorant of it. Our limits will not permit us to enter into any of these interesting points which M. Kilian has passed by unnoticed: we cannot, however, pass by a rather amusing specimen of vague assertion, which at the best must be the result of ignorance:

"Our opinion," says the Professor, "of the English accoucheurs must be very unfavorable, if we are to draw our conclusions of the operator's skill from the form of his instruments; for they use such the chief merit of which is that they can be conveniently concealed in the breeches pocket!"

The absurdity of such a statement is its best refutation. It is a pity that Professor Kilian, when he was in London, did not employ his time to more advantage. The forceps of Denman, Osborne, Clarke, Gooch, Hopkins, besides those of the various accoucheurs of the present day, prove that the Professor is not more acquainted with the modern English forceps than he has shown himself to be with those of former times.

In the enumeration which follows of the more important forceps which have been invented at different times, with their dates, the author fairly owns that Mulder, up to a certain period, has been his authority. We cannot, of course, object to his using this admirable work as a reference; but, when the Professor copies a wrong date, it shows the extent of his reading on this subject, and that Mulder must have been his "ultima Thule" of literary research. He is right to put a mark of interrogation to the date (1725,) of Drinkwater's forceps, because there is strong reason to believe that Drinkwater possessed them much earlier, although not so early as Mulder states, viz. 1668, when, according to Dr. R. W. Johnson, he first entered practice. The date of Dusee's forceps is earlier than 1733; because, after having used them in practice, and made several improvements, he communicated them to Dr. Butter, of Edinburgh, whose description was only published in 1733. Again, 1734 is merely the date of the publication of Giffard's work, which took place after his death. If Prof. Kilian had only read the title-page, he would have seen his error; and, if he had read the book itself, he would have found that

* January 8, 1831. "Historical Analysis of the English Midwifery Forceps."

Giffard used his *extractor* at least as early as 1726. Nor is 1735 the date of Chapman's forceps; because in 1733 (the date of his first edition,) he was evidently in possession of them, but did not choose to divulge them.

The date of Dr. Leake's "Lecture introductory," &c. where he describes his three-bladed forceps, is incorrectly given by Mulder, probably an accidental oversight or misprint, (1774, instead of 1782.) Professor Kilian, we presume, has never referred to Leake's work; otherwise, he would not have copied the wrong date. The date also of Dr. Edward Foster's work is printed 1788 in Mulder, instead of 1781. If the Professor had consulted Froriep's list of obstetric authors, which in other parts of his work he seems to have found particularly useful, he would have there seen the right date. The author regrets (p. 561, *note*,) that Professor Naegelé's highly-praised forceps have not been described: we are not aware that Professor Naegelé has invented any new forceps; the instruments which he uses resemble those of Boer and the late W. J. Schmitt, which are very similar to the Smellie forceps.

It is impossible to follow Professor Kilian in his most diffuse observations on the application and use of the forceps, and on the indications for applying them. We may briefly state, that the forceps acts in three ways; by traction, by compression, and as a lever. As a general rule, we may say that they are indicated in all cases where (the head presenting, and the antero-posterior diameter not less than three inches,) the natural powers are unable to expel the child; or, secondly, where circumstances dangerous to the mother or child require that the labour should be hastened. The rules for applying the forceps are those usually followed in Germany; viz. the patient is placed across the bed, propped up in a half-sitting posture by pillows, &c., her pelvis resting upon the edge, her feet on two chairs, the knees supported by assistants. The female blade, as it is called, is applied first; two, and generally three, fingers are passed, if possible, up to the os uteri, on the side where the blade is to be introduced; the index-finger is held a little behind the middle finger, so that this last, by projecting somewhat, forms a species of ledge on which the blade slides, and which acts as a fulcrum to it; a point which the Professor does not mention. The handle is held at first nearly perpendicular; but, as the blade advances, it gradually approaches the horizontal direction, being guided by the pelvic curve of the instrument. The middle finger, along the ulnar surface of which the convex edge of the blade slides, prevents its extremity from passing too far backwards, and directs it in the axes of the pelvis. When introduced to the full extent, the handle is inclined obliquely downwards, and is now grasped by an assistant, passing his hand below the patient's thigh. The other blade is introduced in the same way on the opposite side of the pelvis; and the locking, extraction, &c. conducted much in the same manner as in England.

We have described the operation as we have seen it performed by the late Professor von Siebold, at Berlin; and certainly nothing could surpass the dextrous gentleness (if we may so express ourselves,) of his operating. This mode of applying the forceps when the patient is lying on her back, we have found useful on more than one occasion.

The ninth Chapter is on Perforation. Professor Kilian uses a trepanshaped perforator; an instrument which we strongly disapprove of, as not fulfilling one of the most important objects of perforation,—viz. splintering the cranial bones. The various lancet-shaped and other cutting perforators of former times, which made the opening at a fontanelle or suture, were ill adapted for the purpose; inasmuch as, when a small quantity of brain had escaped, the bones, coming nearer together, closed the opening. The ring-scalpel, as used by Rœderer, Simpson, &c., and the long curved lancet used by Wigand, in modern times, were exceedingly dangerous instruments, and all subject to the above objection. Professor Joerg, of Leipzig, we believe, is the only practitioner of any rank of the present day who uses the trepan-formed perforator. The instrument used for this purpose by Professor Naegelé, of Heidelberg, is by far the most convenient with which we are acquainted. It is a scissor-shaped perforator, with the point and edges exactly like that of Dr. Denman's, but, instead of the blades crossing at the joint, they merely meet in a hinge, and again diverge; so that, when we press the handles together, the points open. By this means we obtain a great increase of power; for, by merely grasping the handles firmly, we can make the blades diverge, and dilate the opening, without removing the finger of our other hand, which we had placed against the head, to guard the soft parts of the mother. This is not possible with Denman's perforator: the finger and thumb of one hand are not sufficient to open the handles, and we are therefore constrained, after having made the perforation, to use both hands in pulling the handles asunder. This is hazardous; for a sudden movement on the part of the patient may have withdrawn the point of the instrument from the head; and, not having a finger now upon the spot, we cannot be sure that all is safe. In order to keep the points of Naegelé's perforator in close apposition whilst entering the head, the extremities are fixed with a catch, something like that with which the common sugar-nippers is furnished.

The different views which practitioners have entertained as regards this very important operation, have been in many cases so widely different, that it is impossible not to suppose the extreme modes of practice on both sides faulty. The violent and unmeasured opposition which was raised against perforation by the celebrated Osiander, was one among the many peculiar views which were entertained by this learned but extremely opinionated author. Osiander declared, that under no circumstances was perforation justifiable; because in those cases where, according to other practitioners, it was indicated, he could deliver the patient by means of his immensely powerful forceps. Osiander has forgotten to tell us what became of the mother and child after such an operation. To give our readers an idea of how little open to conviction he was on these subjects, we may observe that, when compelled to perforate in cases of hydrocephalus, he then called it *paracentesis capitis.*

If there be one point in obstetric practice more difficult than another, it is to determine the precise limits beyond which the forceps ceases to be applicable, and the perforator becomes indicated. To fix the boundary line merely by ascertaining the degree of pelvic contraction, is obviously incorrect; as this may depend, in great measure, on the size of the child's head, the hardness or softness of the cranial bones, the rigidity of the soft parts, and condition of the mother, &c.: hence it is that we occasionally meet with cases where the perforation is required in a patient who has already borne a living child in her former confinement, and perhaps even without assistance. Nor is it a proof of a practitioner having perforated unnecessarily in her first labour, because in her second the child is born alive, and without any peculiar difficulty. Cases of this sort are well known to occur in large practice: a patient is delivered of her first child alive, and after a perfectly natural labour; in her second, so far from this being easier, she will perhaps require the forceps, and in her third the perforator; and yet the fourth labour will pass over as easily and favorably as the first. These varieties in the same individual depend not merely on one, but on a combination of many causes; among others, none is more worthy of attention than the swelling of the soft parts, especially *below* the head, from interrupted circulation. If the head has been pressing for some time against the pelvis, the soft parts become puffy and œdematous, and, together with the swelling of the cranial integuments of the child, combine to diminish still further the space between the head and pelvis, and increase the difficulty. Dr. Campbell's judicious observations on these points have not been noticed by our author.

Professor Kilian very properly combats the attempt to make a certain degree of pelvic contraction the guide for our use of the perforator, and gives a number of cases from French authors, where living children are said to have been born under circumstances of considerable pelvic deformity. To determine the precise degree of pelvic deformity up to a line, as Madame Boivin professes to do, is, in our humble opinion, rather hazardous; but there can be no doubt but that the head of the child, in some cases, will undergo an extraordinary alteration of shape by the yielding of its bones and overlapping of their edges, without injuring life. The author tells us (p. 746-7,) that, "where the head is fast locked in the contracted pelvis of the mother, so long as her powers are undiminished, and the pains very energetic, we must not think of perforation." When is it that we may venture to wait and see what nature can do to force the head through the contracted pelvis? When the pains are "*very energetic?*" Assuredly not, for we are running a great risk of rupturing the uterus; but when they are steady and moderate, for then the head will have time to yield and adapt itself to the passages.

Chapter X. is on Embryulcia. The author's first indication is, to our notion, little short of nonsense. Embryulcia is indicated "in cases of such pelvic deformity that the Cæsarean section would be indicated, but where the child is evidently dead, and the mother, for the sake of the dead child, will on no account submit to the operation." Under such circumstances, we should say that the patient is much wiser than the doctor. Who in their senses would think of performing a Cæsarean section where the child was known to be dead, and embryulcia not impossible? The name of Dr. Clarke, of Dublin, does not once occur here, nor of course any of his excellent directions for performing this operation, some of which we briefly quoted in our review of Dr. Collins's work, vol. ii. p. 80.

In Chapter XI., on the Cæsarean Operation, nothing worth notice occurs except a most singular (and we are quite certain original,) proposition of the author, viz. that in cases where the Cæsarean section would only be performed where the child is known to be certainly alive, he recommends that we should ascertain this point by rupturing the membranes when the os uteri has dilated, introducing the hand into the uterus, and feeling for the pulsations of the cord. It is singular that Professor Kilian, after the statements made in his first volume, should not have mentioned auscultation as a means for determining this important point, but not a word about the stethoscope occurs.

The indications and directions for performing the operation are minute to a most useless and perplexing degree. There is no doubt but that during the last few years the Cæsarean operation, in the hands of the German practitioners, has lost much of that formidable character which it hitherto possessed. The chief points on which their success has appeared to depend are, operating at an early period of labour, making no useless examinations or attempts to deliver: the incision has been made in the linea alba, the operator has been aided by experienced assistants to support the abdominal parietes either with napkins or sponges dipped in oil, or with the hands alone, and thus all protrusion of the intestines has been prevented. We cannot quote a more instructive case than that of which we gave an extract in our third Number, p. 270, where the Cæsarean operation was performed three times successfully on the same female. The second operation appears to have lasted barely five minutes, and without any considerable suffering on the part of the patient until the moment of making the sutures.

Chapter XII., on the Section of the Pubes, we pass over, as being an operation which has never been practised to any extent, and in the present day would not even be deemed justifiable.

Chapter XIII. considers "The After-Birth Operations;" and, although extending to thirty pages, contains nothing worthy of notice in the present day.

We should here have closed our review of this work; but, to our dismay, a third volume has made its appearance since the first two, and demands our further attention. This gives an account of the purely Surgical Operations of the Accoucheur.

Chapter I. is on the Operation for a Ruptured Perineum. In his introductory remarks, Dr. K. describes the treatment of a common severe case of rupture which heals without artificial assistance, but forgets to say any thing as to the state in which the bowels are to be kept; a circumstance which we hold to be of the highest importance. Within the last few weeks, a case of complete rupture into the rectum occurred to our own notice, in which nothing was done beyond applying cold wet cloths to the wound, keeping the parts very clean by injections into the vagina, and obtaining early and liquid evacuations from the bowels. During the first twenty-four hours the fæces passed through the proper opening, and only once, viz. in about thirty-six hours after the accident, did a small quantity of feculent matter pass away involuntarily through the vagina. The parts gradually contracted, the patient remained very quiet, and complete union followed.

We agree with Wigand that rupture of the perineum heals more rapidly when it takes an oblique direction; and the author's observation, that this form of rupture occurs more frequently than where it runs straight towards the sphincter, agrees entirely with our own experience. Where severe perineal rupture has taken place, most practitioners, both of former and modern times, recommend that the operation should be attempted immediately; whereas a few, among whom are Carus of Dresden, and Dieffenbach of Berlin, consider that this should not be done till at least the month has expired. The author rightly agrees with this latter view, because we never can tell at first, in desperate cases, how far nature will not go towards healing the wound herself, and certain it is that spontaneous cures take place much more frequently than artificial ones. We glean from his account of the operation two points; first, that we should never attempt to unite the edges of the rupture as far as the anterior margin of the perineum, since it is the posterior portion upon the union of which the patient's cure depends; and 2dly, to isolate that portion of the rupture where the suture has been made, on the plan recommended by Dr. Dieffenbach. This consists in making an incision through the skin of the perineum, commencing about 4—6 lines sidewards from the posterior edge of each labium, running in a curved direction, and terminating at about three lines to one side of the anus. By this means the edges of the laceration are not liable to be torn asunder by any movement of the legs, being completely isolated from the neighbouring parts, the edges are brought together by means of the fine pins which Dr. D. uses in the hare-lip and other operations, and are thus placed in the most perfect apposition. Union therefore takes place by the first intention; whereas the incisions on each side, being fresh wounds, are allowed to heal by granulation. The value of these lateral incisions removing tension where parts have been brought together, we have more than once had occasion to admire in the hands of this distinguished operator, and regret that his mode of practice, with a few exceptions, has not excited that degree of interest in this country which it so well deserves.

The bowels are *not* to be rendered costive upon the day of the operation by giving a grain or more of opium, (as Prof. K. recommends,) so that the patient shall have no relief for at least three or four days, but by mild laxatives are to be kept in the gently fluid state we have already mentioned. His dose of calomel and jalap at the end of a four days' constipation is extremely objectionable; except a severe drastic purge, he could not have selected under such circumstances a more unfit combination. Where the circulation of the patient is not below par, we consider the use of cold applications, as recommended by Dr. Dieffenbach, very valuable: we thus restrain the inflammation to the adhesive form, and prevent its proceeding to suppuration or sloughing.

Chapter II. Catheterizing the Female Bladder. The Professor tells us that, wherever we can remove the retention of urine by cataplasms, *leeches*, vapour baths, &c. within due time, we should not resort to the catheter. In most cases, it is true, we should try warm fomentations, sitting over steams of hot water, &c.; but as to *leeches*, we think that passing a catheter is a far shorter and simpler operation. The degree of distention which the bladder has attained is a more important guide for passing the catheter, than retention for a longer or shorter time, because involuntary dribbling of urine to a considerable quantity is frequently observed where the bladder is distended to a dangerous degree. We feel bound to meet with a flat contradiction the author's random assertion, that the English practitioners place a female on her side, with her knees drawn up very high, for the purpose of passing the catheter. Like Professor K., we have always recommended the elastic catheter; and in all cases of difficulty, whether from pressure or displacement, have found it infinitely preferable to the silver one. It is far safer in the hands of a midwife.

M. Kilian's rules for finding out the position of the orificium urethræ, are precisely those which we ourselves have practised for many years, although we make no claim to originality, having received them from an eminent practitioner since deceased. He very properly directs the operator to feel for the cushion-like prominence, which the urethra forms beneath the symphysis pubis, and following this to its anterior extremity, he comes without fail to the orificium. We would have quoted his words, but his verbosity prevents us. He mentions in a note that in England a bladder is frequently attached to the end of the catheter to receive the urine; a common wine-bottle is more convenient, as it may be emptied several times, if necessary, without disturbing the catheter.

Where there has been considerable distention of the bladder, we frequently find, after the urine has ceased to flow, that, on passing a catheter a little further, we come, as it were, into another cavity from which we succeed in drawing off a large quantity, owing probably to incomplete contraction of the bladder.

Chapter III. is on the Operation for Imperforate Vagina and External Parts, but contains nothing deserving of notice.

Chapter IV. is on the Operation for Prolapsus Uteri. Professor K. recommends that, where the prolapsus resists all our attempts to reduce it, we should use bloodletting and the warm bath. The former, where there is no febrile action to indicate its use, will not be justifiable here, and we should rather suspect that the latter remedy would, if any thing, aggravate the evil. If he had understood the pathology of prolapsus uteri, he would have found that active leeching and afterwards cooling applications, would in such cases have been more effectual. Where a prolapsed uterus has become very large and heavy, local detraction of blood is almost necessary in every case; not only with regard to the mere reduction, but also mainly as respects the retention of it afterwards. We have repeatedly seen cases of prolapsed uterus, which had resisted the common remedies, completely relieved by the self-same treatment when reduced in size and weight by leeching. In the following page (83,) another observation occurs, which proves to our mind that the author has but an imperfect and confused view of the subject. He affirms that local corroborant remedies are of far more value than general ones. Nothing can be more incorrect than such an opinion. Without the use of medicines to improve the general health, local remedies, of whatever sort they may be, will produce but little good. Every practitioner of any experience will accord with us in saying that many cases of partial prolapsus will recover entirely, where improvement of the general health has been carefully attended to, without the application of local remedies. On the other hand, we own that in some cases of long standing prolapsus, where the health is but little, if at all, affected, the use of local astringents will form the chief part of our treatment.

Dr. K. asserts that vaginal injections are of no use in prolapsus uteri. In this opinion we cannot agree with him. When combined with the necessary attention to the general health, we believe them always a most valuable adjuvant in the cure; but we fear they are often very injurious in the hands of some practitioners, who appear to trust to them almost exclusively.

Professor Kilian recommends the medicated pessary, (a cylindrical bag filled with some vegetable astringent powder,) mentioned in our last Number in the review of Dr. Hamilton's work; but even this is far inferior, we think, to a piece of soft sponge rolled into a cylindrical form, wound spirally round with twine to prevent its expanding, and soaked in an astringent mixture. A mere sponge introduced without this preparation does more harm than good, as it acts like a common tent, and dilates the vagina still further; and we ought to have stated more explicitly in our remarks on this subject in our last Number (p. 134), that we object almost as much as Dr. Hamilton to the use of the ordinary pessaries on this account: at least, if they do not actually dilate the vagina, they prevent its contraction.

Professor Kilian describes the Operative Treatment of Prolapsus Uteri *during Labour.* The circumstances under which this may occur are not mentioned. It occurs where the pains are unusually severe, and accompanied by most violent detrusive efforts. In many instances, an exceedingly rapid labour is the result; but in other cases, where the os uteri does not yield so rapidly, the whole uterus will be forced low down into the pelvis, so that even the lower third or half will protrude at the os externum. The treatment in moderate cases will consist in merely desiring the patient to resist the impulse to strain as far as lies in her power; to remove all towels, &c. to pull by, or cushions to push against; to place the pelvis high, and shoulders low; and to keep her as cool and quiet as possible. Where the os uteri is considerably detruded before it is fully dilated, and even projects at the os externum, all attempts to support it with the hand, as recommended by the author, are utterly useless. This will only irritate and inflame the os uteri, and thus delay its dilatation. Where matters have come to this extent, the only method which will be of any avail is that recommended by Professor Naegelé, viz. of putting on a strong broad T bandage, and cutting a slit through it where it corresponds to the os externum. The child's head will press against and ultimately force its way through this opening, while the uterus is prevented from descending any further. Professor Naegelé tried this plan with perfect success in a case where in the former labour the lower half of the uterus had protruded, and where the pains this time were equally violent.

Chapter V. Operation for Inversion of the Uterus. That this displacement will be attended with very different effects in different cases, is an observation of the author's with which we perfectly agree; that in some cases the most alarming symptoms or even death will occur; that in others the patient will experience little or no derangement. The reasons of this variety he has not given, nor do Dr. Dewees's excellent observations on inversion appear to have excited his attention. The severity of the symptoms depends on the degree of inversion. In partial inversion, where the fundus or a portion of it has passed through the os uteri, where in fact the uterus is in a state of intussusception, and tightly girt by the spasmodically contracted os uteri, the most terrible and alarming symptoms will result. In the words of Dr. Dewees, "the woman almost instantly complains of a severe and distressing pain about the region of the uterus; an effort to force or bear down, nausea, and sometimes vomiting; great faintness, with more or less hemorrhage, cold clammy sweats, pulse small, frequent, or extinct." The reduction is easier in partial, but the danger greater than in complete, inversion. In the latter the uterus, it is true, is turned wrong side outwards, but there is no strangulation, and being capable of contracting, there is much less danger from hemorrhage. And here we differ in some degree from the opinion of Sir C. M. Clarke, where he says that "it is generally, if not always, a consequence of mismanagement of the placenta;" and are supported in our opinion by the experience of Dr. Dewees. "It has been almost universally supposed," says this excellent author, § 1303, "that an undue force applied to the cord for the delivery of the placenta was the principal cause of this accident; but in this we differ from such authorities as have adopted the opinion, and for the following reasons: First, because the accident has occurred after the delivery of the placenta. Second, because it has taken place when no such force has been applied; but the caution of not applying too much force to the cord for the withdrawing of the placenta is founded upon just and important principles; since, did the disposition to inversion exist, and this mass be attached to the fundus, it would be almost certain to produce it, when perhaps without such force the woman might escape from the danger."

Cases frequently occur where the uterus is but slightly contracted after the birth of the child, and where, on pulling the cord, we feel the fundus so moveable that it seems as if a moderate degree of force would be sufficient to bring it down. On the other hand, several cases have occurred to us where the patient has been suddenly surprised with a violent pain, and the child expelled before she could reach her bed or even lie down on the floor. In at least three of these cases the cord was broken, and thus gave evidences of considerable force having been applied to the fundus. Where the fundus is not reducible, Professor Kilian recommends that we should keep it in the vagina by a proper pessary, and, to diminish its size still further, to scarify the surface of the uterus, and to rub in iodine, mercurial or cicuta ointment, strict diet, &c. We scarcely know how to reconcile such a recommendation as this with any degree of practical knowledge in the proposer. To rub in ointments of this sort on the surface of a chronically inverted uterus with a profuse leucorrhœal discharge, or perhaps a menorrhagic hemorrhage, every ten or fifteen days; for the cure of which, moreover, under such a debilitating drain to the system, she is put on strict diet, &c.!!

In cases where it is very difficult or impossible to return an inverted uterus, the Professor recommends compressing it with the hands in order to diminish its bulk. This plan was practised successfully by Mr. C. White, of Manchester, many years ago, and M. Kilian ought to have mentioned the circumstance. The case to which Mr. White alludes was one of nearly complete inversion, which had occurred about an hour before his arrival. Finding that the taxis did not succeed, but brought on violent forcing, accompanied with profuse hemorrhage, and as the body of the uterus appeared too large to pass through the os uteri, which was contracted, "I grasped," says Mr. White, "the body of it in my hand, and held it there for some time, in order to lessen its bulk by compression; as I perceived that it began to diminish, I persevered, and soon after made another attempt to reduce it by thrusting at its fundus, it began to give way, I continued to force it until I had perfectly returned it, and had insinuated my hand into its body. It was no sooner reduced than the pulse at her wrist began to beat; she recovered as fast as we could wish." In addition, Professor Kilian recommends active bloodletting, warm bath, tobacco injections, poultices with belladonna, &c. injections with warm oil, and lastly, if the os uteri be still rigid (or rather we should say if the woman be still alive), incisions into it, and blames the "ridiculous fears" of the French and English practitioners for not trying this mode of practice! We need hardly say how decidedly improper this practice is.

Chapter VI. Operation for Retroversion of the Uterus. This chapter occupies fifty-seven pages, and contains an immense quantity of literature jumbled together in a very confused manner. The names of Dewees and Burns are, it is true, mentioned, but none of their valuable observations quoted: however, as the views of both these authors on retroversion are remarkably clear and valuable, we recommend them to our younger readers. An appendix follows on anteversion, or, as he calls it, pronation of the uterus. The Professor considers this an extremely rare displacement.

Retroversion occurs most frequently in the gravid state, anteversion in the unimpregnated state, and has appeared to us, in those cases which we have seen, to be connected with gastric or menstrual derangement. The prominent symptoms are, the not being able to retain much water in the bladder, a sense of pressure and pain about the pubes, with dragging in the groins. In severe, or rather we should say complete cases, the os uteri will be found turned directly to the sacrum, and a firm solid body, viz. the fundus, pressing against the neck of the bladder. We have once only met with it during early pregnancy, and by pressing up the tumour which was close behind the symphysis pubis, we were enabled to reach the os uteri sufficiently to hook it down with the finger into its natural position.

Chapter VII. Operation for Vesico-Vaginal Fistula. Professor Kilian has collected a considerable quantity of literature upon this highly important subject. Much may be done to diminish this injury, were the patient able in every case to lie upon her side with the catheter in the bladder, as soon as it is discovered that the water comes by the vagina; for, by keeping the bladder constantly empty, and preventing the urine from escaping through the opening, the parts contract so much that the wound will either close of itself, or diminish so considerably as to be capable of being cured by caustic or actual cautery. At all events this plan should always be premised, before having recourse to the operation itself. In many cases, however, so much fever and abdominal inflammation have been brought on by the same cause which has produced the local injury, that it is impossible to attend to these points until the patient herself is in a state of safety. The vesico-vaginal fistula, with a view to the operation, may be looked upon as of two sorts, the longitudinal and the transverse. We can scarcely look upon those cases where the posterior wall of the cervix vesicæ has been entirely destroyed as curable, although the bold and skilful attempts of Dr. Dieffenbach have proved successful under circumstances the most discouraging. Professor Naegelé in his "Erfahrungen und Abhandlungen," published 1812, was one of the first to propose a plan for curing this hitherto irremediable mischief. One method was by means of a peculiar forceps, which, being furnished with minute points or prickles, was enabled to seize the edges of the opening, which had been previously pared for the purpose, and keep them in close apposition until union was effected. This instrument is only adapted for longitudinal openings, although we think that a modification, if necessary, might easily be made for those which are transverse. Where there is a considerable loss of substance it will be but of little use. Under these circumstances, the results of Dr. Dieffenbach's practice, detailed in his surgical observations, are exceedingly valuable, and show that union may be effected under circumstances which, with any other mode of treatment, would have been hopeless.

Professor Naegelé has also proposed a plan of making the suture *per urethram*, but merely mentions the idea in his observations on this subject, and, as far as we know,, never carried it into effect. It has, however, been tried with perfect success by Dr. Koehler, and a description of the operation given in his Hospital Reports, a periodical work full of valuable surgical practice, published in Polish at Warsaw. Dr. K. uses a curved catheter open at the extremity, and furnished with a *porte-aiguille* or needle-holder: having pared the edges, he introduces the forefinger of his left hand into the vagina against one of them, and directing the point of the catheter, which he holds in his right hand, to the corresponding part on the vesical side of the opening, transfixes it with the needle which the catheter contains; this (previously armed with a long ligature) is seized by a forceps per vaginam, and brought out at the os externum. The other end of the ligature is now attached to a needle, which is fixed as before in the *porte aiguille*, and passed into the catheter as the first was, the finger per vaginam and the catheter per urethram are directed to the corresponding portiòn of the opposite edge of the opening, and thus the other end of the ligature is brought through the os externum. The ligature is tightened by a proper instrument (*serre-nœud*:) and the operation repeated according to the number of sutures required. We have not as yet seen this operation performed, but, from its great simplicity, and from the assurances which Dr. K. gave us of its success, have no hesitation in recommending it to the notice of our countrymen.

In Chapter VIII. on the Operation for Polypus of the Vagina and Uterus, nothing occurs worthy of noting, although it extends to fifty pages. A great number of different instruments for tying polypi are both described and delineated: no notice, however, is taken either of Gooch's or Gräfe's polypus instruments, which are by far the most valuable.

Chapter IX. on the Operation for Partial or Total Extirpation of the Uterus, is, like most of the other parts of the work, diffuse and tedious, and contains nothing of novel interest.

No mention is made throughout this work of applying leeches to the os and cervix uteri. We are the more anxious to call the attention of our readers to this point, because we stated in one of our former Numbers, (Review of Duparque, No. IV.) that the practice had scarcely been tried in this country; a statement, as we have since learned, which is not correct. We ought to have confined our observation to the published records of practice. We now find that the application of leeches to the os uteri has been pretty extensively used in private practice, not only in the metropolis, but in many other parts of England; and, as we have ascertained, with much benefit. This remedy is indeed regarded by those who have used it as one of the greatest improvements in this branch of practice. Inflammation of the cervix uteri, generally of a subacute character, is by no means an unfrequent disease among females, and is but too often followed by scirrhous induration, lancinating pains, and carcinomatous destruction. The application of leeches to the os and cervix uteri, repeated according to circumstances, and aided by the exhibition of medicines calculated to regulate and promote the general health of the system, is the mode of treatment which offers the greatest chance of success when once this insidious disease has established itself. Four or five leeches thus applied will produce more relief than several ounces of blood abstracted by cupping from the sacrum, loins, &c. This is of great importance; for, not only in the above-mentioned affection, but also in several derangements of the menstrual function, where leeches to the os uteri have been attended with great success, there has been too much debility to warrant any considerable depletion. The mode of application by means of a straight tube with holes at its extremity, is, we presume, too well known to require any description.

---

